# The India Face Set: International and Cultural Boundaries Impact Face Impressions and Perceptions of Category Membership

**DOI:** 10.3389/fpsyg.2021.627678

**Published:** 2021-02-11

**Authors:** Anjana Lakshmi, Bernd Wittenbrink, Joshua Correll, Debbie S. Ma

**Affiliations:** ^1^Department of Psychology, University of Chicago, Chicago, IL, United States; ^2^Booth School of Business, University of Chicago, Chicago, IL, United States; ^3^Department of Psychology and Neuroscience, University of Colorado Boulder, Boulder, CO, United States; ^4^Department of Psychology, California State University, Northridge, Los Angeles, CA, United States

**Keywords:** normed face stimuli, India and U.S., cultural differences, subjective impressions, stereotypes

## Abstract

This paper serves three specific goals. First, it reports the development of an Indian Asian face set, to serve as a free resource for psychological research. Second, it examines whether the use of pre-tested U.S.-specific norms for stimulus selection or weighting may introduce experimental confounds in studies involving non-U.S. face stimuli and/or non-U.S. participants. Specifically, it examines whether subjective impressions of the face stimuli are culturally dependent, and the extent to which these impressions reflect social stereotypes and ingroup favoritism. Third, the paper investigates whether differences in face familiarity impact accuracy in identifying face ethnicity. To this end, face images drawn from volunteers in India as well as a subset of Caucasian face images from the Chicago Face Database were presented to Indian and U.S. participants, and rated on a range of measures, such as perceived attractiveness, warmth, and social status. Results show significant differences in the overall valence of ratings of ingroup and outgroup faces. In addition, the impression ratings show minor differentiation along two basic stereotype dimensions, competence and trustworthiness, but not warmth. We also find participants to show significantly greater accuracy in correctly identifying the ethnicity of ingroup faces, relative to outgroup faces. This effect is found to be mediated by ingroup-outgroup differences in perceived group typicality of the target faces. Implications for research on intergroup relations in a cross-cultural context are discussed.

## Introduction

It has been noted that psychology conducts its research largely on people from Western, educated, industrialized, rich, and democratic countries—coined WEIRD societies by Henrich et al. (2010). Social psychology, despite its focus on the importance of social context for psychological functioning, is no exception in this regard. Within the area of intergroup relations, studies on stereotyping, group attitudes, and intergroup behavior, have been conducted largely with participants from the United States and Western Europe, investigating how people perceive, judge, and interact with social groups that are culturally relevant to these parts of the world. By comparison, studies with participants and/or target groups from non-WEIRD societies are few and far between (e.g., Jahoda, 1959; Kashima et al., 2003; Cuddy et al., 2009; Durante et al., 2017). The limited empirical scope raises questions about how research findings might generalize to other cultural contexts. And it leaves the field with missed opportunities for studying psychological determinants of intergroup relations.

Ironically, recent efforts to improve methodological practices in psychology (Kahneman, 2012; Asendorpf et al., 2013; Open Science Collaboration, 2017) carry some risk to further exacerbate this situation. For example, in order to improve experimental control and to facilitate comparisons across studies, researchers are encouraged to rely on standardized procedures and materials in their studies (Shrout and Rodgers, 2018). However, such standardization is likely to come at the expense of methodological diversity. A case in point is the Chicago Face Database (CFD; Ma et al., 2015), a collection of face images and norming data that our lab developed and made available as a free resource for use as stimulus materials in research.

The database provides easy access to face images that are uniform in terms of image quality, lighting, camera positioning, model pose, and other potentially confounding aspects of photographs. The face images come with extensive norming data that cover physical attributes (e.g., face height, width, luminance, etc.) as well as subjective impressions of the faces (e.g., perceived age, attractiveness, trustworthiness, etc.), allowing researchers to select images for particular face attributes while controlling for other factors that are extraneous to the research question. The database was inspired by the International Affective Picture System (IAPS; Lang et al., 1997), a stimulus database that has seen widespread use in research involving emotion and affect. Similar to the IAPS, the CFD was intended to facilitate and help standardize the broad variety of psychological research that involves the presentation of face stimuli to participants (e.g., impression formation, intergroup processes, stereotyping, prejudice, emotions). Since its release just 5 years ago, the database has seen rapid adoption, with more than 7,000 downloads and 700 published papers that report studies with CFD faces.

An explicit goal in developing the database was also to broaden the demographic composition of face images available to researchers. The existing image resources available include either exclusively Caucasian faces (Ekman and Friesen, 1976; Troje and Bülthoff, 1996; Lundqvist et al., 1998), or only a relatively small number of non-Caucasian faces (Tottenham et al., 2009; Langner et al., 2010; DeBruine and Jones, 2017; see Table 1 for a list of widely used image sets and their ethnic makeup). In contrast, the CFD now offers images and norming data for nearly 600 Asian, Black, Latino, and White males and females.

**TABLE 1:** Face image sets and their ethnic makeup.

Database	Number of models by ethnicity	
CFD-India	Indian Asian	142
CFD (Ma et al., 2015)	Asian	109
	Black	197
	Caucasian	183
	Latino	108
FACES (Ebner et al., 2010)	Caucasian	171
KDEF (Lundqvist et al., 1998)	Caucasian	70
Facelab London Set (DeBruine and Jones, 2017)	Asian	19
	Black	13
	Caucasian	69
	Multiethnic	1
NimStim (Tottenham et al., 2009)	Asian	6
	Black	10
	Caucasian	25
	Latino	2
POFA (Ekman and Friesen, 1976)	Caucasian	14
RaFD (Langner et al., 2010)	Caucasian	39
	Moroccan	18

While the database makes it easier for researchers to include non-Caucasian faces in their studies, all CFD models were volunteers recruited in the U.S. As a result, the ethnic diversity represented in the database remains limited to a subset of U.S. ethnic social groups. And the composition of these groups reflects the obvious limitations of a convenience sample. For instance, models of the database who self-identified as *Asian* are predominantly U.S.-born models with East Asian ancestry, covering only a portion of the ecological diversity of faces on the Asian continent. Likewise, the subjective norming data included in the database were collected with U.S. rater samples. They offer information on how attractive etc. the faces appear to U.S. participants, but raise the question whether these impressions might be different for perceivers of different cultural background and/or group identity.

While researchers have employed creative methods such as morphing and caricatures to generate additional non-Caucasian face stimuli from the limited number of available base faces (e.g., Byatt and Rhodes, 1998; Krumhuber et al., 2015), the focus of existing face databases on Caucasian faces has obvious methodological and conceptual implications. The reliance on U.S.-specific norms for stimulus selection may introduce experimental confounds if the norms don’t generalize to non-U.S. participants. The use of face stimuli that insufficiently capture the ecological diversity of faces may adversely impact a study’s external and internal validity (Wells and Windschitl, 1999) and yield incorrect effect estimates or fail to identify important moderators (Fiedler, 2011). In addition, the ready availability of certain ethnicities in the database may influence what target groups are being chosen for investigation in the first place, curtailing research on hypotheses for which materials aren’t readily available.

### The Current Research

The research reported in this paper aims to address some of these issues and improve the usefulness of the database for work with non-U.S. participants and non-U.S. faces. It has three specific goals. First, we describe the development of an expansion to the CFD with face images of individuals recruited in India, drawing on a large non-U.S. ethnic group that accounts for approximately 18% of the world population (United Nations Department of Economic and Social Affairs Population Division, 2019). Second, we explore the extent to which subjective impressions of these faces are culturally dependent. And, third, we investigate whether differences in target face familiarity and perceived group typicality impact judgments of face ethnicity.

#### The India Face Set

The new image set introduced here includes high resolution face images of 142 unique individuals, displaying a variety of facial expressions (neutral, angry, fearful, and happy). The images are standardized according to the procedures used for the CFD and, hence, can serve as stimuli side-by-side with the original U.S. face images. They are accompanied by comprehensive norming data. Beyond the physical face attributes and subjective impressions that are part of the CFD, these norms now also include self-reported background information on the models (e.g., ancestry, home state, religious affiliation, caste, and SES measures). All materials are available as a free resource at www.chicagofaces.org.

#### Cultural Dependency of Subjective Image Norms

A second goal of the current research is to explore the extent to which the subjective rating norms are culturally dependent and the extent to which these ratings might differ for ingroup and outgroup faces. Although some studies have found impressions from faces to be consistent across culturally diverse rater samples (Wagatsuma and Kleinke, 1979; Bernstein et al., 1982; Cunningham et al., 1995) several recent studies have documented systematic cultural differences in what impressions perceivers glean from faces (Sutherland et al., 2018; Wang et al., 2019; Jones et al., 2021). Moreover, there are various theoretical arguments and related empirical findings that would suggest impressions for ingroup faces and outgroup faces to differ. For example, the mere exposure hypothesis (Zajonc, 1968) predicts that more familiar faces should be judged more positively. In fact, faces with feature sets near the population average are perceived to be more familiar (Langlois et al., 1994). And familiar faces, in turn, are judged as more likable (Zebrowitz et al., 2008), trustworthy (Lewicki, 1985), and attractive (Winkielman et al., 2006; Zebrowitz, 1996). To the extent that Indian and U.S. faces differ systematically in their feature sets, and that raters are relatively more familiar with their ingroup, one would expect ingroup faces to be viewed more positively. Theories of intergroup behavior, such as social identity theory (Tajfel et al., 1979), would similarly predict impressions to reflect ingroup favoritism, with impressions of ingroup faces to be more positive.

On the other hand, social stereotypes may also impact impressions of both ingroup and outgroup faces with regard to particular stereotypic attributes. For example, the stereotype content model suggests that groups viewed as competitors are perceived to be less warm, and groups of lower status as less competent (Fiske et al., 2002). With regard to the groups of interest to the current research, Lee and Fiske (2006) observed that U.S. participants’ stereotypes of Indian Asian immigrants are similar in content and valence to the stereotypes U.S. participants hold about their own ingroup. Also, though we are unaware of any direct data on this issue, a 2014 Pew Research Center Survey suggests that the majority of Indians hold favorable (58%) or very favorable (30%) views of the U.S. (Pew Research Center, 2014). Based on these data we might expect impression ratings to reflect mutual admiration, rather than ingroup favoritism.

To explore these possibilities, we collected subjective impression ratings in a full ingroup-outgroup design, with samples of Indian and U.S. participants each rating both Indian Asian and Caucasian face images on a variety of attributes (e.g., attractiveness, competence, etc.). The design allowed us to identify separate effects of participant and target group on face impressions, and test for evidence of stereotyping and ingroup/outgroup favoritism in these ratings.

#### Judgments of Face Ethnicity

Finally, a third goal of the research was to determine whether differences in familiarity with Indian and Caucasian faces would impact participants’ ability to identify face ethnicity. Across domains, stimulus familiarity has been found to impact processing efficiency (Posner and Keele, 1968; Lewellen et al., 1993) and categorization (e.g., Smith, 1967; Johnson and Mervis, 1997; Whittlesea and Leboe, 2000). In the case of faces, it has been suggested that familiar ingroup faces function as a perceptual default facilitating their processing and identification, while impeding the processing and identification of other-race faces (e.g., Goldstein and Chance, 1980; Rhodes et al., 1987; Macron et al., 2009). Hence we expected greater accuracy in judgments of familiar faces, with Indian Asian faces to be more likely classified as such by Indian raters than U.S. raters, whereas the opposite should hold for Caucasian faces.

## Materials and Methods

### Face Stimuli

The present study used Caucasian and Indian Asian target faces as experimental stimuli. Caucasian face stimuli were randomly drawn from the existing pool of CFD images depicting Caucasian models from the U.S. (for a full list of target images, see the online Supplementary Material). Face stimuli for Indian Asian targets were collected at the University of Chicago Center in Delhi, India. Potential volunteers were contacted via convenience sampling, snowball sampling as well as pamphlets that were distributed to various cultural organizations with memberships from different regions in India. Volunteers were required to be between the ages of 18 and 50. Of the resultant volunteers, 53 were female and 91 were male. Self-report data about the volunteers’ location within India (87 North Indian, 15 South Indian, 15 West Indian, 12 North East Indian, 7 Central Indian, 7 East Indian), religion (79 Hindu, 25 Muslim, 19 Sikh, 18 Christian, 1 Jain, 1 agnostic, 1 no religion), caste category, native language, education, employment and annual income were collected as was information about location of birth, current location of residence and ancestry.

#### Photo Sessions

Upon arrival participants were each asked to carefully read an informed consent and image release form. The forms were made available in both English and Hindi, and upon request were translated on site to other Indian languages. For illiterate participants, the experimenter read aloud the consent instructions and probed for comprehension. Afterward, participants changed into a gray t-shirt (the same type of shirt worn by all models of the existing CFD image set). Next, at the participants’ discretion, they removed any make-up and jewellery. If needed, they were encouraged to shave and adjust their hair so that it did not obstruct the face. We chose not to enforce compliance with these grooming preparations as they may have interfered with cultural practices. For example, some married women in India wear vermillion on the apex of their hairline and/or a traditional necklace. Tradition may prevent them from appearing in public without these signifiers of their married status. Likewise, men may grow a beard or wear a turban for religious purposes. In such instances, volunteers were photographed as is.

For the actual photo session, volunteers were then seated at a fixed distance from a digital camera. The technical setup for these sessions followed closely the procedures used for the existing CFD image set, described in detail in Ma et al. (2015). Volunteers were asked to make neutral, happy (with both open and closed mouth smile), angry and fearful expressions while also maintaining an upright and straight head position. Each volunteer completed three rounds of photographs. In the first round, they received a prompt (e.g., “make a closed mouth smile”), and when necessary, the photographer followed up with more specific directions (e.g., “Please try to engage your eyes in the smile”). The second and third round repeated the full cycle of facial expressions. Volunteers who struggled reaching credible expressions were offered illustrations taken from Ekman and Friesen (1976). This resulted in multiple photographs for each volunteer displaying each of the requested facial expressions. Sessions lasted approximately 30 min. At the conclusion, refreshments were provided and thereafter volunteers were thanked and compensated with Rs. 500.

#### Image Standardization

From the resulting pool of images, we selected one neutral expression image per volunteer, based on head position (i.e., straight and upright) image quality (i.e., in focus), and how neutral the expression indeed was. Using these criteria, two (for a subset of targets, three) independent judges first rated each image and identified their top three face stimuli. Next, these top picks were used to settle on a consensual best choice for the final image selection^1^.

**FIGURE 1:**
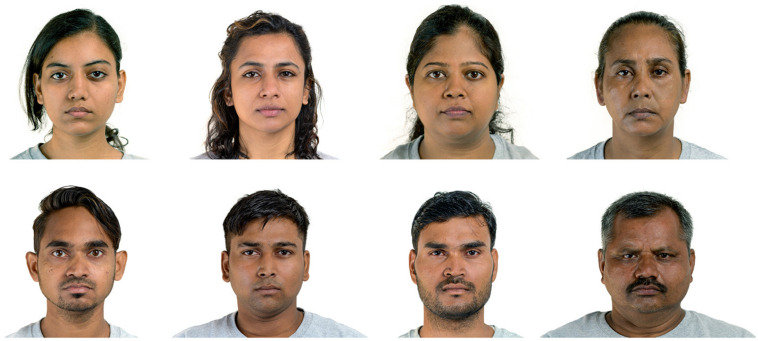
Sample Stimuli from the India Face Set.

The selected images were edited using Adobe Photoshop software (version 20) following the standardization procedures described in Ma et al. (2015). RAW image files were corrected for uniform color temperature and exposure across images, matching the existing CFD materials. Where necessary, additional corrections were made to reach a realistic skin tone. Next, we made digital modifications to select images, to remove any blemishes, markings or tattoos, facial or ear piercings, as well as any earrings, hair accessories and/or jewelry^2^. All images were then resized so that the size of the core facial features was more or less equivalent across all images and consistent with the existing CFD face stimuli. For this, a 796 pixels (wide) × 435 pixels (high) template was fit over the target’s core facial features, adjusting the image size such that either the eyebrow-lip distance matched the template height, and/or the max. cheekbone distance matched the template width. Finally, a white background was inserted, and the image was exported to a 2,444 pixels by 1,718 pixels JPEG file (see Figure 1 for sample images).

### Norming Data

The standardized neutral expression images serve as the basis for the norming data, which include both objective measures of physical face features and subjective ratings of face impressions. The latter are the focus of our question whether subjective image norms are culturally dependent. In contrast, the objective norms are part of the image set development and provide descriptive information on the physical attributes of the new face sample. We report them here in order to document the steps we took to capture the physical attributes of the India face set.

#### Subjective Norms

Subjective ratings of the 284 target faces (142 Indian Asian, 142 Caucasian) with neutral facial expressions were obtained using two separate tasks (A and B) designed with Qualtrics Research Suite Software. Task A asked Indian and U.S. participants to rate the Indian Asian and Caucasian faces on a range of attributes. In task B a separate sample of Indian and U.S. participants was asked to rate the Indian Asian and Caucasian target faces for their group typicality. Participant recruitment and data collection for both tasks were conducted using Amazon’s Mechanical Turk.

##### Task A

In a within-subjects design, each participant was presented with 8 target faces—2 Caucasian male, 2 Caucasian female, 2 Indian Asian male, and 2 Indian Asian female. For each participant, these 8 faces were selected at random from the target pool, with no replacement until all of the target faces were judged once for that iteration. The entire task took approximately 15 min to complete; U.S. participants were compensated with $3 and Indian participants with Rs. 100.

For each target, participants first saw the target pictured at the top of the computer screen followed by prompts below to estimate the target’s age, race (with response options: Chinese Asian, Japanese Asian, Indian Asian, Other Asian, Black, Hispanic/Latino, White/Caucasian, and Other) and gender. Next, the target image remained, but the prompts were replaced, asking participants to rate their impression of the target on the following dimensions: attractive, warm, competent, trustworthy, happy, sad, disgusted, surprised, fearful/afraid, angry, threatening, masculine, feminine, baby-faced, and unusual (such that they would stand out in a crowd). For each target, these attributes were presented across two successive screens and the ordering of attributes within each screen was chosen at random. Participants responded with a Likert scale of 1 (Not at all) through 4 (Neutral) to 7 (Extremely). The next screen showed a prompt asking participants to characterize the social status of the target from 1 (Low) through 7 (High). To facilitate these ratings, the prompt was accompanied by the following explanation: People of high status are typically thought to be wealthy and well-educated, working in highly paid jobs whereas those who are of low status are thought to be poor and not well-educated (or not educated at all), typically working in low paid positions or unemployed (see Lakshmi et al., 2019). All items but for status, competence and warmth were drawn from Ma et al. (2015). Status, warmth and competence were included to assess any evidence of stereotyping as suggested by the Stereotype Content Model (Fiske et al., 2002).

In addition to these items, Indian participants received several additional prompts that were omitted for U.S. participants as the queries required more detailed knowledge of Indian culture. Specifically, Indian participants were asked to further estimate the ethnicity of each Indian Asian target (with response options: North Indian, South Indian, North East Indian, East Indian, West Indian, Anglo Indian, and Other), their caste category (upper, middle, lower and tribe) and their religious affiliation (Hindu, Muslim, Christian, Sikh, Buddhist, Jain, Jewish, Parsi/Zoroastrian, No religion, and Other). These data are not of interest to the current study but are available via the norming data distributed with the CFD-India face set.

We took several steps to ensure data quality. Participants completed a bot check (captcha) and a Geo-IP check at the start of the task. The Geo-IP check filtered for participant IP addresses to be located either in India or the United States while excluding participants connected via a Virtual Private Network (VPN) to mask their country location. Following the Bot/Geo-Ip check, the actual survey began with an instructional manipulation check (IMC; Oppenheimer et al., 2009). This IMC was intended to screen out random clicking participants. It consisted of a set of instructions at the top of the screen, followed by a Likert scale with items labeled 1 through 9, and an arrow at the bottom of the screen. Instructions asked participants to advance to the next screen by clicking on the arrow and to ignore the scale items.

1,709 Indian participants and 2,937 U.S. participants offered consent and cleared the bot check. Of these, 1,226 Indian participants and 1,839 U.S. participants passed the Geo-IP test and completed their task. Of these participants, 981 (80%) Indian participants and 1,371 (75%) U.S. participants responded accurately to the attention check, suggesting similar data quality in the India and U.S. samples. Of these, 878 Indian participants (238 female, average age = 33.51, age sd = 8.48) and 900 U.S. participants (392 female, average age = 37.61, age sd = 11.39) self-reported as Asian Indian and White/Caucasian, respectively, and had no missing data in their records.

##### Task B

For this second task, we divided the 284 target faces into four subsets along target gender and ethnicity: Indian Asian females, Indian Asian males, Caucasian females, and Caucasian males. In a between-subjects design with face subset as the between-participant factor, each participant was presented with 40 target faces chosen at random from one of these four face subsets. The entire task took about 10 min to complete; U.S. participants were compensated with $2 and Indian participants with Rs. 70.

Depending on the experimental condition, participants were instructed that they would see pictures of Indian Asian males (Indian Asian females; Caucasian males; Caucasian females). The instructions further explained that these people would differ in terms of how much their physical features resemble the features of Indian (White) people. For example, their skin color, hair, eyes, nose, cheeks, lips, and other physical features, may be more Indian/White (i.e., typical of Indians/White people) or less Indian/White (i.e., less typical of Indians/White people). Their task would be to rate how Indian (White) looking each person’s physical features were. Thereafter participants saw Indian Asian (Caucasian) male (female) targets one at a time, and rated how typical that person’s physical features are of Indian (White) people. They were offered a 5-point scale (less typically Indian (White) looking, somewhat typically Indian (White) looking, fairly typically Indian (White) looking, more typically Indian (White) looking and very typically Indian (White) looking.

Participants also completed the same set of bot check (captcha), Geo-IP check, and ICM used in task A. Given the screen layout and response format stayed consistent in this task, rather than switch from screen to screen as in task A, we included a second attention check. For this check, the very last target trial displayed a female Latino target face with the word “Less” superimposed on the forehead. Instructions asked participants to select the response option that matched the word displayed on the face.

339 Indian and 594 U.S. participants offered consent and cleared the bot check. Of these, 335 Indian and 459 U.S. participants also cleared the Geo-IP check. 260 (78%) Indian and 348 (76%) U.S. participants responded accurately to the first attention check, of which 218 Indian and 276 U.S. participants also responded accurately to the second attention check, again indicating similar data quality in the India and U.S. samples. Among this participant set, 215 Indian participants (51 female, average age = 31.35, age sd = 7.46) and 207 U.S. participants (91 female, average age = 37.19, age sd = 12.02) self-reported as Asian Indian and White/Caucasian, respectively and completed the entire task.

#### Objective Norms

For the Caucasian faces included in the current study, measurements of the physical features are available as part of the existing CFD norming data. For the Indian Asian face stimuli, we carried out physical measurements in accordance with the procedures described in Ma et al. (2015). Table 1 in the Supplementary Material summarizes all measures and the calculations used to obtain them. In response to requests from researchers, and because the literature in some cases has used multiple definitions for a given measure, the objective norms have been expanded since the original release of the database. We included the full expanded set of physical norms in our assessment of the Indian Asian face stimuli. Specifically, the following measurements were obtained: median luminance of the face, nose width, nose length, lip thickness, face length, height and width of each eye, face width at the most prominent part of the cheek, face width at the mouth, face width at the ears, forehead length, distance between each pupil and the top of the head, distance between each pupil and the upper lip, distance between pupils, chin length, length of cheek to chin for both sides of the face, the distance between the middle of each brow and the hairline atop that brow, face color (red, green, blue), hair color (red, green, blue), thickness of each eyebrow and eyelid. Using the CFD measurement guide (available on the database website), three coders independently completed the measurements in Adobe Photoshop. For each face and measure, the coders’ average measurements were computed and individual measurements that exceeded the mean by 20% in either direction were flagged. These differences were then discussed and reconciled by the research team (consisting of the three coders, joined by A.L., and B.W.) A final set of measures was obtained based on the resulting raters’ averages. The inter-rater reliability for these measurements was acceptable to high (Cronbach’s alpha equaled 0.69 on face width at cheeks, and was between 0.72 and 0.99 on all other attributes).

## Results

### Subjective Norms

Our analyses focus on the subjective impression and ethnic classification ratings. Specifically, these analyses address two questions with regard to how the participant sample (India vs. U.S.) may have impacted ratings of the target faces: (1) do the resulting stimulus norms for the target groups vary with the participant sample, and if so, do these differences reflect stereotyping and/or ingroup favoritism? (2) do perceptions of face ethnicity vary with participant sample, such that categorization accuracy is higher for ingroup than outgroup targets? Across analyses, participant and target group were each contrast coded (0.5 = Indian/Indian Asian, −0.5 = U.S./Caucasian)^3^.

#### Impression Ratings

We first considered whether the subjective stimulus norms varied with participant sample and whether any of these differences varied with target group, across impression attributes. Next, we examined the specific effects on individual impression attributes. For the overall effect, we ran a linear mixed effects model using the lme4 package(Bates et al., 2015) in R with participant impression ratings as the dependent variable and participant group and target group as independent variables. In this analysis, attribute ratings were standardized within each attribute, and then averaged across attributes per participant per target. Participant and target face were included as random effects variables. The full set of results from this analysis is available inSupplementary Material. We focus here on the participant group main effect and the target group by participant group interaction. There was no significant main effect of participant group (*p* = 0.722) but there was indeed a significant interaction effect between participant and target group [*t*_(__12188.4__)_ = −7.71, *p* < 0.001, η^2^*_*p*_* = 0.005 (0.00, 0.01)]. Across attributes, both Indian participants [Caucasian faces: Mean z score = 0.621 vs. Indian Asian faces: Mean z score = −0.749; t_(__12188.4__)_ = −9.28, *p* < 0.001] and U.S. participants [Caucasian faces: Mean z score = 0.359 vs. Indian Asian faces: Mean z score = −0.233; t_(__12188.4__)_ = −3.89, *p* < 0.001] gave higher impression ratings for Caucasian faces than Indian Asian faces, however this effect was significantly higher among Indian participants.

To clarify how participant group impacted each of the impression attributes, and whether any of these differences varied with target group, we conducted separate linear mixed effects models for each attribute (using the same model specifications as in the parent model, but scores were not standardized within attribute since we were not combining data across attributes for these analyses). Means and test statistics for the participant group and target group main effects are reported in Table 2 (see the online Supplementary Material for a complete set of test statistics). In these analyses, participant group had a main effect on impression ratings for happiness, anger, surprise, fear, masculinity, babyface, competence and perceived status. Indian, compared to U.S. participants rated the target faces to be more happy [*t*_(1769_._9__)_ = 3.24, *p* = 0.001, η^2^*_*p*_* = 0.006 (0.00, 0.01)] and less angry [*t*_(1770_._2__)_ = −2.89, *p* = 0.004, η^2^*_*p*_* = 0.005 (0.00, 0.01)]. Several impression attributes showed target group main effects: Indian Asian faces were judged to be less babyfaced [*t*_(__278_._8__)_ = −7.58, *p* < 0.001, η^2^*_*p*_* = 0.170 (0.11, 0.24)] and more unusual [*t*_(__251_._2__)_ = 3.10, *p* = 0.002, η^2^*_*p*_* = 0.040 (0.01, 0.08)] than Caucasian faces. In addition, participant group by target group interactions emerged for attractiveness, competence, trustworthiness, anger, masculinity, babyfacedness, unusualness, and status. A breakdown of these interactions is reported in Table 2. Next, we explored whether these observed effects reflected any systematic pattern of stereotyping and/or ingroup favoritism.

**TABLE 2:** Attribute ratings by participant group and target group.

Attribute	Effect means and standard deviations			
Attractive	Participant × Target***	All Ps	India Ps**	U.S. Ps**
	All Targets***	4.07_(__1_._62__)_	4.04_(__1_._65__)_	4.09_(__1_._59__)_
	India Targets**	3.88_(__1_._62__)_	3.79_(__1_._65__)_	3.96_(__1_._58__)_
	U.S. Targets	4.26_(__1_._60__)_	4.30_(__1_._61__)_	4.21_(__1_._60__)_

Warm	Participant × Target	All Ps	India Ps	U.S. Ps
	All Targets	3.88_(__1_._58__)_	3.87_(__1_._58__)_	3.89_(__1_._58__)_
	India Targets	3.86_(__1_._58__)_	3.84_(__1_._58__)_	3.88_(__1_._58__)_
	U.S. Targets	3.90_(__1_._57__)_	3.91_(__1_._57__)_	3.90_(__1_._57__)_

Competent	Participant × Target***	All Ps***	India Ps*	U.S. Ps
	All Targets	4.37_(__1_._43__)_	4.15_(__1_._53__)_	4.58_(__1_._30__)_
	India Targets**	4.36_(__1_._45__)_	4.11_(__1_._56__)_	4.61_(__1_._29__)_
	U.S. Targets**	4.38_(__1_._42__)_	4.20_(__1_._50__)_	4.55_(__1_._32__)_

Trustworthy	Participant × Target*	All Ps	India Ps	U.S. Ps
	All Targets	4.33_(__1_._42__)_	4.32_(__1_._47__)_	4.34_(__1_._38__)_
	India Targets	4.34_(__1_._42__)_	4.30_(__1_._46__)_	4.37_(__1_._38__)_
	U.S. Targets	4.32_(__1_._42__)_	4.33_(__1_._47__)_	4.32_(__1_._38__)_

Happy	Participant × Target	All Ps**	India Ps**	U.S. Ps*
	All Targets **	3.37_(__1_._75__)_	3.47_(__1_._73__)_	3.28_(__1_._77__)_
	India Targets*	3.25_(__1_._73__)_	3.33_(__1_._70__)_	3.17_(__1_._75__)_
	U.S. Targets**	3.50_(__1_._77__)_	3.60_(__1_._74__)_	3.40_(__1_._79__)_

Angry	Participant × Target*	All Ps**	India Ps	U.S. Ps
	All Targets	2.85_(__1_._78__)_	2.76_(__1_._72__)_	2.93_(__1_._83__)_
	India Targets**	2.87_(__1_._77__)_	2.76_(__1_._70__)_	2.97_(__1_._83__)_c
	U.S. Targets*	2.83_(__1_._78__)_	2.76_(__1_._73__)_	2.90_(__1_._83__)_c

Sad	Participant × Target	All Ps	India Ps*	U.S. Ps
	All Targets*	3.20_(__1_._82__)_	3.23_(__1_._81__)_	3.17_(__1_._82__)_
	India Targets	3.31_(__1_._82__)_	3.35_(__1_._82__)_	3.26_(__1_._82__)_
	U.S. Targets	3.09_(__1_._80__)_	3.11_(__1_._79__)_	3.08_(__1_._82__)_

Disgusted	Participant × Target	All Ps	India Ps	U.S. Ps*
	All Targets	2.76_(__1_._75__)_	2.77_(__1_._69__)_	2.76_(__1_._82__)_
	India Targets	2.79_(__1_._76_	2.79_(__1_._68__)_	2.80_(__1_._83__)_
	U.S. Targets	2.74_(__1_._75__)_	2.76_(__1_._69__)_	2.71_(__1_._81__)_

Surprised	Participant × Target	All Ps***	India Ps**	U.S. Ps**
	All Targets***	2.71_(__1_._77__)_	2.91_(__1_._72__)_	2.52_(__1_._81__)_
	India Targets**	2.65_(__1_._75__)_	2.83_(__1_._70__)_	2.47_(__1_._79__)_
	U.S. Targets**	2.78_(__1_._79__)_	2.98_(__1_._73__)_	2.57_(__1_._83__)_

Fearful	Participant × Target	All Ps***	India Ps	U.S. Ps*
	All Targets	2.81_(__1_._75__)_	2.95_(__1_._71__)_	2.68_(__1_._79__)_
	India Targets**	2.86_(__1_._76__)_	2.98_(__1_._71__)_	2.74_(__1_._79__)_
	U.S. Targets**	2.77_(__1_._75__)_	2.92_(__1_._71__)_	2.62_(__1_._78__)_

Threatening	Participant × Target	All Ps	India Ps	U.S. Ps
	All Targets	2.96_(__1_._79__)_	2.98_(__1_._75__)_	2.93_(__1_._84__)_
	India Targets	2.96_(__1_._79__)_	2.98_(__1_._74__)_	2.94_(__1_._84__)_
	U.S. Targets	2.95_(__1_._8__)_	2.98_(__1_._76__)_	2.93_(__1_._83__)_

Masculine	Participant × Target*	All Ps***	India Ps	U.S. Ps
	All Targets	3.84_(__2_._07__)_	3.75_(__2_._08__)_	3.93_(__2_._06__)_
	India Targets**	3.89_(__2_._10__)_	3.78_(__2_._11__)_	4.01_(__2_._09__)_
	U.S. Targets*	3.80_(__2_._04__)_	3.73_(__2_._05__)_	3.86_(__2_._02__)_

Feminine	Participant × Target	All Ps	India Ps	U.S. Ps
	All Targets	3.81_(__2_._17__)_	3.81_(__2_._21__)_	3.80_(__2_._13__)_
	India Targets	3.70_(__2_._19__)_	3.71_(__2_._24__)_	3.70_(__2_._13__)_
	U.S. Targets	3.91_(__2_._15__)_	3.91_(__2_._17__)_	3.91_(__2_._13__)_

Babyfaced	Participant × Target**	All Ps***	India Ps**	U.S. Ps**
	All Targets***	3.14_(__1_._84__)_	2.94_(__1_._81__)_	3.34_(__1_._85__)_
	India Targets**	2.92_(__1_._81__)_	2.69_(__1_._74__)_	3.16_(__1_._84__)_
	U.S. Targets**	3.37_(__1_._85__)_	3.20_(__1_._84__)_	3.53_(__1_._84__)_

Unusual	Participant × Target***	All Ps	India Ps*	U.S. Ps**
	All Targets**	3.09_(__1_._79__)_	3.13_(__1_._75__)_	3.04_(__1_._82__)_
	India Targets	3.14_(__1_._78__)_	3.09_(__1_._75__)_	3.19_(__1_._82__)_
	U.S. Targets**	3.04_(__1_._79__)_	3.18_(__1_._76__)_	2.90_(__1_._82__)_

Status	Participant × Target***	All Ps***	India Ps**	U.S. Ps**
	All Targets***	4.42_(__1_._31__)_	4.58_(__1_._30__)_	4.27_(__1_._29__)_
	India Targets**	4.17_(__1_._32__)_	4.26_(__1_._33__)_	4.09_(__1_._31__)_
	U.S. Targets**	4.67_(__1_._24__)_	4.89_(__1_._19__)_	4.45_(__1_._26__)_

All cell values are Means_(SD)_; Main Effects indicated at “All Targets”/“All Participants” cells (for Target and Participant main effects respectively); Interaction effects indicated at “Participant × Target” cells; Simple effects indicated at “India Ps”/“U.S. Ps”/“India Targets”/“U.S. Targets” cells;*p ≤ 0.05.**p ≤ 0.01.***p ≤ 0.001.

##### Stereotyping

Here we considered the ratings for the basic stereotype dimensions suggested by the Stereotype Content Model (SCM; Fiske et al., 2002; also see Kervyn et al., 2015), warmth, trustworthiness, and competence. In our analyses, stereotyping could be evidenced as a target group main effect, such that participants from both India and the U.S. differentiate Indian Asian from Caucasian faces in similar fashion. Alternatively, Indian and U.S. participants could stereotype their respective ingroup and outgroup differently, resulting in a participant by target group interaction. While analyses for the competence ratings showed no significant target group main effect (*p* = 0.800), a significant main effect of participant group, qualified by a significant interaction effect between target group and participant group emerged [*t*_(12199_._4__)_ = −4.45, *p* < 0.001, η^2^*_*p*_* = 0.002 (0.00, 0.00)]. Simple slopes analyses reveal that U.S. participants rated Indian Asian faces (Mean = 4.61, SD = 1.28) as marginally more competent than Caucasian faces [Mean = 4.55, SD = 1.32; *t*_(12199_._4__)_ = 1.75, *p* = 0.080]. On the other hand, Indian participants, rated Caucasian faces (Mean = 4.20, SD = 1.50) as significantly more competent than Indian Asian faces [Mean = 4.11, SD = 1.56; *t*_(12199_._4__)_ = −2.19, *p* = 0.030; see Table 2]. Trustworthiness ratings showed no significant main effect of target group or participant group, but again, yielded a significant interaction effect between target group and participant group, with the respective outgroup faces being seen as more trustworthy (Indian participants: Mean = 4.33, *SD* = 1.47; U.S. participants: Mean = 4.37, *SD* = 1.38) than the ingroup (Indian participants: Mean = 4.30, SD = 1.46; U.S. participants: Mean = 4.32, *SD* = 1.38) ratings [t_(12185_._9__)_ = −2.20, *p* = 0.028, η^2^*_*p*_* = 0.0004 (0.00, 0.00); see Table 2]. Simple slopes analyses for comparing the target group means within participant group were not significant (all *p*s > 0.270). No significant effects emerged for perceived warmth (all *p*s > 0.141).

Group perceptions of competence have reliably been found to correlate with and be informed by perceived social status (Fiske et al., 1999; Caprariello et al., 2009), suggesting that ratings of perceived social status should parallel our results for competence. In fact, the analyses for perceived status do yield this target group by participant group interaction [*t*_(12170_._4__)_ = −8.28, *p* < 0.001, η^2^*_*p*_* = 0.006 (0.00, 0.01)]. However, the pattern of means deviates somewhat from the results for perceived competence. Simple slopes analyses indicate that, while both participant groups rated Caucasian faces higher in status, this effect was greater among Indian participants [Caucasian faces: Mean = 4.89, *SD* = 1.19 vs. Indian Asian faces: Mean = 4.26, *SD* = 1.33; *t*_(12170_._4__)_ = −11.41, *p* < 0.001] than U.S. participants [Caucasian faces: Mean = 4.45, *SD* = 1.26 vs. Indian Asian faces: Mean = 4.09, *SD* = 1.31; *t*_(12170_._4__)_ = −6.34, *p* < 0.001; see Table 2]. Given this pattern, correlations between perceived competence and status remain modest (*r* = 0.31).

In summary, our analyses for participants’ ratings of warmth, competence, and trustworthiness show no overall target group differences for warmth, competence, or trustworthiness. However, we do observe differentiation in the impressions of Indian and U.S. Caucasian faces between the two participant groups. For competence and trustworthiness, both participant groups rated the respective outgroup somewhat higher than their own ingroup.

##### Ingroup favoritism

The second question we posed regarding the impression ratings is whether participants would see ingroup targets overall more favorably than outgroup targets. The results for perceived competence and trustworthiness we just summarized would suggest that if anything the current data show the reverse pattern, with outgroup faces receiving more favorable ratings than ingroup faces, on these attributes. In order to address this question more systematically, we calculated two scores to capture the favorability of the impressions: a positivity score using the ratings from all positively valenced impression attributes (attractive, warm, competent, trustworthy, happy; Cronbach’s α = 0.81) and a negativity score with the ratings of all negatively valenced attributes (angry, sad, disgusted, fearful, threatening; Cronbach’s α = 0.86). We calculated a difference score (positivity score—negativity score) as an indicator of impression favorability (Wittenbrink, 2007). We then analyzed these favorability scores in a linear mixed effects model using the lme4 package in R to analyze the data with the favorability scores as the dependent variable and target group and participant group as independent variables. We employed random intercepts for participant and target face stimulus.

The full set of results from this analysis is available in the online Supplementary Material. We focus here on ingroup favoritism, which is represented by the target group and participant group interaction. The effect was small but significant [*t*_(12171_._5__)_ = −2.24, *p* = 0.025, η^2^*_*p*_* = 0.0004 (0.00, 0.00)]. For U.S. participants, impressions of ingroup faces were marginally more favorable than their impressions of outgroup faces [Ingroup faces: Mean = 1.23, SD = 1.73; Outgroup faces: Mean = 1.05, SD = 1.71; *t*_(12171_._5__)_ = −1.81, *p* = 0.070]. For Indian participants on the other hand this pattern reversed. Impressions of outgroup faces were significantly more favorable than their impressions of ingroup faces [Ingroup faces: Mean = 0.90, *SD* = 1.67; Outgroup faces: Mean = 1.16, *SD* = 1.76, *t*_(12171_._5__)_ = −2.87, *p* < 0.001).

##### Typicality

A final impression item asked participants to rate the target faces in terms of group typicality. We first examined the effects of target group and participant group on perceived target typicality. Analyses of these typicality ratings employed the same mixed effects model used for all other impression attributes. With regard to effects involving participant group, these analyses yielded a significant main effect—Indian participants (Mean = 3.46, *SD* = 1.18) rated typicality overall higher than U.S. participants [Mean = 3.24, *SD* = 1.28; *t*_(15902_._2__)_ = 12.87, *p* < 0.001, η^2^*_*p*_* = 0.010 (0.01, 0.01)]. This main effect was qualified by a significant interaction effect between participant group and target group [*t*_(15902_._2__)_ = 12.84, *p* < 0.001, η^2^*_*p*_* = 0.010 (0.01, 0.01)]. Simple slopes analyses indicate that Caucasian faces (Mean = 3.41, *SD* = 1.29) were perceived as significantly more typical than Indian Asian faces (Mean = 3.04, *SD* = 1.24) by U.S. participants [*t*_(15902_._2__)_ = −6.15, *p* < 0.001] but the same difference did not emerge for Indian participants (Caucasian faces: Mean = 3.41, *SD* = 1.14; Indian Asian Faces: Mean = 3.51, *SD* = 1.22; *p* = 0.140). Unrelated to our question of interest, there was also a significant main effect of target group on perceived typicality—Caucasian faces (Mean = 3.41, *SD* = 1.22) received higher typicality ratings overall than Indian Asian faces [Mean = 3.28, *SD* = 1.25; *t*_(__270_._3__)_ = −2.45, *p* = 0.015, η^2^*_*p*_* = 0.020 (0.00, 0.06)].

### Face Categorization

Our second primary research question concerned perceptions of face ethnicity for the two target groups and whether they would vary with participant sample. Because of greater familiarity with ingroup faces, we expected participants to more accurately identify the ethnicity of their respective ingroup faces.

#### Categorization Accuracy

To address this question we calculated for each target face the probability of accurate categorization as a proportion of the number of times the target face was categorized correctly (i.e., an Indian Asian face identified as Asian Indian, and a Caucasian face judged to be Caucasian), relative to the number of times it was categorized at all. The resulting accuracy score served as the dependent variable in a binomial generalized linear model using the lme4 package (Bates et al., 2015) in R with target group and participant group as independent variables. Weights were added to the model based on categorization count; i.e., the number of times each target was categorized at all.

**FIGURE 2:**
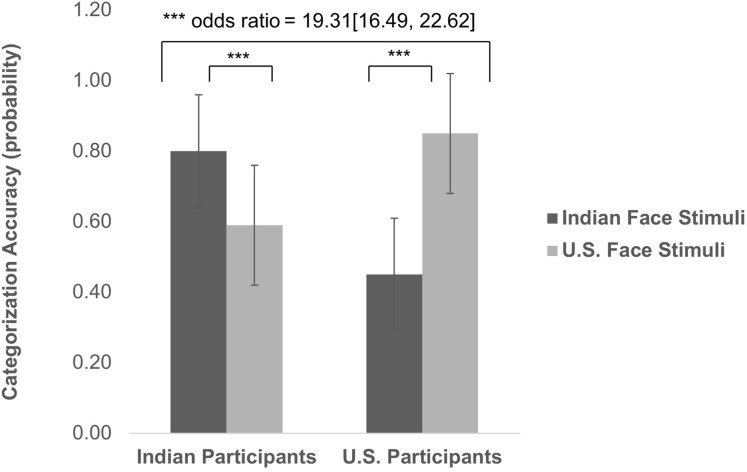
Ingroup-Outgroup Differences in Categorization Accuracy.

Consistent with the expected ingroup accuracy advantage, there was a significant interaction effect between target group and participant group [*t*_(__543__)_ = 36.69, *p* < 0.001, odds ratio = 19.31 (16.49, 22.62); see Figure 2]. Simple slopes analyses of the interaction between target group and participant group indicate that among U.S. participants, the probability of accurate categorization was significantly higher for Caucasian faces (Mean = 0.85, *SD* = 0.17) than for Indian Asian faces [Mean = 0.45, SD = 0.16; *t*_(__543__)_ = −34.23, *p* < 0.001]. Indian participants showed a similar ingroup accuracy bias. For them, the probability of accurate categorization was significantly higher for Indian Asian faces (Mean = 0.80, *SD* = 0.17) than for Caucasian faces [Mean = 0.59, *SD* = 0.16; *t*_(__543__)_ = 17.09, *p* < 0.001]. Unrelated to our primary question, there was a significant main effect of target group [t_(__543__)_ = −13.39, *p* < 0.001, odds ratio = 0.58 (0.54, 0.63)]: Categorization accuracy was overall higher for Caucasian faces (Mean = 0.72, *SD* = 0.21) than Indian Asian faces (Mean = 0.62, *SD* = 0.24).

#### Typicality

Another factor that might impact the categorization of faces is their ethnic typicality. That is, one might expect faces that are seen to be more typically Indian in appearance to be more readily classified as Indian Asian. In fact, such effects of typicality on categorization are well established. A robin is more readily recognized as a bird than an ostrich (Rosch, 1973). Face categorization, including categorization by ethnicity, is no exception and is similarly sensitive to typicality effects(Maddox and Gray, 2002; Locke et al., 2005). We therefore used the typicality impression ratings we already reported earlier to test whether the observed ingroup advantage in categorization accuracy is mediated by perceptions of typicality prevalent in the two participant groups.

For ingroup advantage in categorization accuracy to be mediated by perceived typicality, two conditions have to be met: (1) typicality should affect categorization accuracy (a test of the link between perceived typicality and categorization accuracy); and (2) ingroup-outgroup differences in typicality should affect ingroup outgroup differences in categorization accuracy (a test of the link between ingroup advantage, perceived typicality and categorization accuracy; see Judd et al., 2001).

For each target, we calculated a mean typicality rating and categorization accuracy score for ingroup participants, as well as a mean typicality rating and categorization accuracy score for outgroup participants. Using these measures, we obtained four values for each target: average typicality rating (across ingroup and outgroup), average categorization accuracy (across ingroup and outgroup), difference in typicality rating (ingroup–outgroup) and difference in percentage accuracy(ingroup–outgroup). Using these scores, we set up two linear models to test the influence of group membership and typicality ratings on categorization accuracy.

**FIGURE 3:**
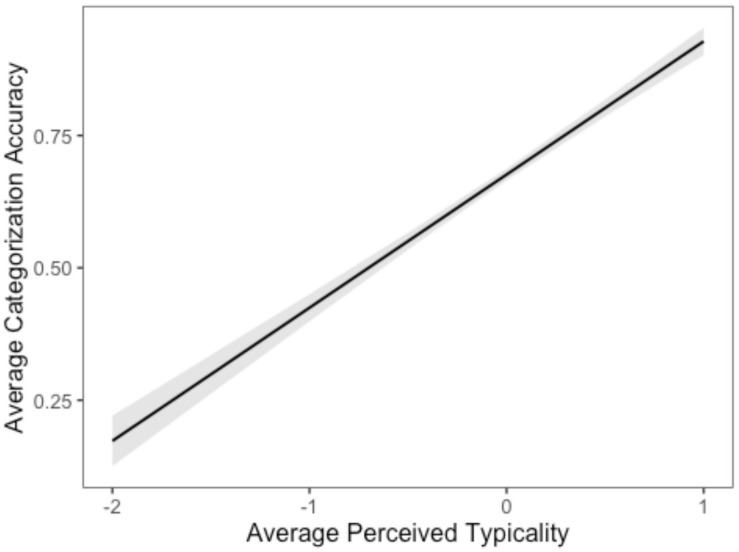
Average Perceived Typicality and Categorization Accuracy.

The first linear model used average categorization accuracy as dependent variable and mean centered average typicality as independent variable. There was a significant intercept [Mean = 0.68, *t*_(__270__)_ = 119.30, *p* < 0.001] suggesting that on average, categorization accuracy was significantly above zero controlling for typicality. Average typicality added significantly to categorization accuracy [*t*_(__270__)_ = 21.24, *p* < 0.001, η^2^ = 0.630 (0.57, 0.67)], with categorization accuracy improving with higher perceived average typicality (see Figure 3). In other words, average typicality did affect categorization accuracy.

**FIGURE 4:**
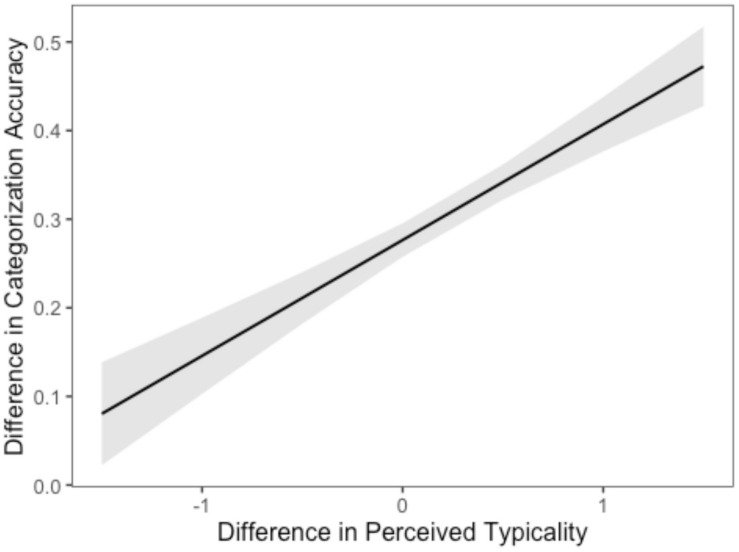
Ingroup-Outgroup Differences in Perceived Typicality and Categorization Accuracy.

In the second linear model, difference in categorization accuracy served as the dependent variable and the ingroup/outgroup difference in average typicality ratings served as the independent variable. There was a significant intercept Mean = 0.28, *t*_(__270__)_ = 28.55, *p* < 0.001, suggesting that on average, ingroup/outgroup difference in categorization accuracy was significantly above zero, controlling for ingroup/outgroup difference in average typicality. Ingroup-outgroup difference in mean typicality ratings added significantly to the ingroup-outgroup difference in categorization accuracy. As the ingroup-outgroup difference in mean rating increased, so did the ingroup-outgroup difference in categorization accuracy [*t*_(__270__)_ = 7.96, *p* < 0.001, η^2^ = 0.190 (0.12, 0.26); see Figure 4].

Thus, these analyses suggest that the effect of familiarity (as determined by group membership) on categorization accuracy was mediated significantly albeit not fully, by perceived typicality.

#### Miscategorization

Finally, we explored what categories were used in error when Indian Asian faces were not identified as Indian Asian, and Caucasian faces not judged to be Caucasian. Toward this, we selected all instances of inaccurate categorizations and identified the two most common ethnicities participants chose in these instances, Middle Eastern and Hispanic/Latino, accounting for 54.65% of all erroneous categorizations. For each target face, we then generated percentages of inaccurate categorization as (1) Middle Eastern and (2) as Latino, separate for Indian participants and U.S. participants, respectively. For example, to calculate the percentage of inaccurate categorization as Middle Eastern, the number of times a target was inaccurately categorized as Middle Eastern was divided by the number of times it was inaccurately categorized at all. As with accurate categorizations, we employed a binomial generalized linear model using the lme4 package (Bates et al., 2015) in R to analyze the data with probability of inaccurate categorization as Middle Eastern and Latino, respectively, as the dependent variables and target group and participant group as independent variables, weighted by target categorization count.

For inaccurate categorization into Middle Eastern, there was a significant effect of target group such that Indian Asian faces (Mean = 0.31, *SD* = 0.26) were inaccurately categorized as Middle Eastern with higher probability than Caucasian faces were [Mean = 0.21, *SD* = 0.22; *t*_(__504__)_ = 1.97, *p* = 0.049, odds ratio = 1.18 (1.00, 1.38)]. However, this main effect was qualified by a significant interaction between target group and participant group [*t*_(__504__)_ = −5.32, *p* < 0.001, odds ratio = 0.42 (0.30, 0.58)]: For both participant groups, errors made for outgroup faces were more likely to be misjudged as Middle Eastern, compared to ingroup faces with errors. Simple slopes analyses indicate that that U.S. participants inaccurately categorized Indian Asian faces (Mean = 0.42, *SD* = 0.23) as Middle Eastern at a significantly higher probability than they did Caucasian faces [Mean = 0.22, *SD* = 0.28; *t*_(__504__)_ = 5.33, *p* < 0.001]. Indian participants on the other hand, categorized Caucasian faces (Mean = 0.21, *SD* = 0.15) as Middle Eastern at a significantly higher probability than they did with Indian Asian faces [Mean = 0.18, *SD* = 0.23; *t*_(__504__)_ = −2.29, *p* = 0.020].

For inaccurate categorization into Latino, there was a significant main effect of target group such that Caucasian faces (Mean = 0.46, *SD* = 0.30) were inaccurately categorized as Latino more often than were Indian Asian faces [Mean = 0.19, *SD* = 0.20; *t*_(__504__)_ = −14.12, *p* < 0.001, odds ratio = 0.31 (0.27, 0.37)]. The main effect was again qualified by a significant target group and participant group interaction [*t*_(__504__)_ = −2.47, *p* = 0.014, odds ratio = 0.67 (0.48, 0.92)]. Indian participants inaccurately categorized Caucasian faces (Mean = 0.36, *SD* = 0.20) as Latino at a significantly greater probability than they did Indian Asian faces [Mean = 0.10, *SD* = 0.16; *t*_(__504__)_ = −10.51, *p* < 0.001). U.S. participants as well, inaccurately categorized Caucasian faces (Mean = 0.58, SD = 0.35) as Latino at a significantly greater probability than they did Indian Asian faces, but his effect was smaller than among Indian participants [Mean = 0.27, *SD* = 0.20; *t*_(__504__)_ = −9.47, *p* < 0.001].

Interestingly, and related to our main research questions with respect to the effect of participant group, we also observed a significant main effect of participant group such that U.S. participants (Mean = 0.32, *SD* = 0.28) inaccurately categorized faces as Middle Eastern at a significantly higher probability than Indian participants did [Mean = 0.20, *SD* = 0.19; *t*_(__504__)_ = −7.71, *p* < 0.001, odds ratio = 0.53 (0.45, 0.62)]. Also, U.S. participants (Mean = 0.42, *SD* = 0.32) inaccurately categorized faces as Latino at a significantly higher probability than Indian participants did [Mean = 0.24, *SD* = 0.22; *t*_(__504__)_ = −12.83, *p* < 0.001, odds ratio = 0.35 (0.30, 0.41)].

## Discussion

Human faces are an important factor in social life. Perceivers use them for a wide range of social inferences about emotions, personal identity, social category membership, traits, preferences, and even culpability in legal cases (e.g., Ekman et al., 1972; Blair et al., 2004; Zebrowitz and Montepare, 2008; Todorov et al., 2015). As a result, a good part of social psychological research involves the presentation of face stimuli. The Chicago Face Database (CFD) is a frequently used resource for this type of work. Since its release just 5 years ago, the database materials have been retrieved by over 7,000 researchers worldwide and some 700 published papers have reported studies with CFD faces. Yet, as is the case with psychological research in general, the database materials remain limited in their cultural and ethnic diversity. Not only by name, the database to-date is U.S.-centric. It contains the faces of volunteers recruited in the U.S., and its stimulus norms are based on U.S. rater samples.

With the current research we set out to broaden the scope of the database and improve its usefulness for work with non-U.S. participants and non-U.S. faces. To this effect, we introduce a new set of face stimuli representing a 142 individuals from a large non-U.S. ethnic group, Indian Asians. We report the development and standardization of these stimulus materials, which follow the established procedures of the database, so that the new Indian Asian images can be used interchangeably with the full set of CFD stimuli. With the new image set, we also provide extensive norming data that cover both the physical face attributes as well as subjective impressions of the faces. Finally, in addition to the neutral expression images relevant to the current research questions, the India face set also includes images of models making a variety of emotional expressions.

The empirical part of the current research then focused on the subjective face impressions included in the norming data. First, we asked whether the resulting face norms are culturally dependent and will vary with the participant sample. To address this issue, we collected impression ratings in a full ingroup-outgroup design with samples of Indian and U.S. participants, for both Indian Asian and Caucasian face images. Results show that impression ratings indeed varied significantly with participant group. Compared to U.S. raters, Indian participants judged faces to be more happy, surprised, fearful, and of higher social status, but less angry, masculine, babyfaced, and competent. The current results add to evidence from other recent studies that impressions from faces are to some extent culturally specific (Sutherland et al., 2018; Wang et al., 2019; Jones et al., 2021). Possibly of greater consequence for the use of these impression norms in selecting or weighting study materials, the differences between participant groups depended on the target group. For example, Indian and U.S. participants significantly differed in their ratings of Indian Asian and Caucasian faces on perceived trustworthiness. Consequently, a study among Indian participants with both Indian Asian and Caucasian faces that relied on U.S. image norms in selecting faces of similar trustworthiness would run the risk of confounding trustworthiness and face ethnicity. Hence, the current findings highlight the importance of obtaining local stimulus norms for research with non-U.S. participant samples.

We further explored whether the differences we observed between Indian and U.S. raters followed systematic patterns of ingroup favoritism and stereotyping. With regard to ingroup favoritism, we observed that U.S. participants reported marginally more favorable impressions for faces of their ingroup, compared to outgroup faces. However, Indian participants’ ratings, in contrast, showed outgroup favoritism. Their impressions of outgroup faces were significantly more favorable than their impressions of ingroup faces. The result highlights the importance of conducting research on intergroup relations across diverse cultural and international settings. While the literature has generated a long history of findings demonstrating general ingroup favoritism in social judgment (Brewer, 2007), our results for the Indian participant sample clearly deviate from this established effect.

With regard to stereotyping, we focused on face ratings of warmth, competence, and trustworthiness, following the SCM by Fiske and colleagues (Fiske et al., 2002; Kervyn et al., 2015). Overall, the results show no target group differences along the basic stereotype dimensions of the SCM. However, we did observe differentiation between the two participant groups. For competence and trustworthiness, both participant groups rated the respective outgroup somewhat higher than their own ingroup. The results may reflect the fact that, for both target groups, outgroup stereotypes are overlapping with ingroup stereotypes. Consistent with this interpretation, Lee and Fiske (2006) found U.S. stereotypes about Indian immigrants living in the U.S. to be largely similar to ingroup stereotypes. In a cluster analysis of stereotype content, Indian immigrants appeared in the same cluster as various ingroups (e.g., college students). Arguably, this study investigated a specific subset of Indian Asians, Indian immigrants in the U.S. However, we should note that in our study the impression rating task (task A) made no reference to the targets’ nationality, ethnicity, or any other social category for that matter. Participants merely saw faces. Without mentioning an international context, it seems quite likely that our U.S. participants considered both the Indian Asian faces as well as the Caucasian faces to represent individuals living in the U.S. Similarly, we suspect our Indian participants considered the Indian Asian faces to depict individuals from their immediate environment, India. Caucasian faces, in contrast, are considerably less prevalent in Indian society and may be more readily assumed to be non-Indian foreigners by our Indian participants. Possibly, differences in attributions between the participant groups with regard to the targets’ background may account for our results for perceived status. Our data deviate somewhat from prior findings, which generally show substantive correlations between perceived group status and perceptions of competence. However, the existing research here has generally focused on status differences within a given society (e.g., Durante et al., 2017).

The relative prevalence of stereotypic impressions for the two target groups may have been further impacted by participants misclassifying face ethnicity. As a matter of fact, as we had predicted categorization accuracy differed significantly for ingroup and outgroup faces. Moreover, the categories chosen most frequently in error differed for ingroup and outgroup faces. In these instances of misclassification, where, for example, an Indian Asian target is seen to be Middle Eastern, we would expect different stereotypes to impact the impression ratings. The observed misclassification of outgroup faces may have considerable real-world consequences, for example in forensic settings where law enforcement officers may use either explicitly or implicitly a suspect’s ethnicity. Likewise, some research suggests that, post 9/11, South Asians living in the United States experienced misclassification as Middle Eastern, resulting in identity threat, stereotyping, and prejudice (Joshi, 2006; Bhatia, 2008; Poolokasingham et al., 2014).

Arguably, there is considerable value in research on group-level stereotypes; research that investigates the content and the dynamics of beliefs about entire groups. And, given the scarcity of data on the stereotypes Indians hold about people from the U.S. and vice versa, we wish more of this kind of group-level research was conducted in an international context.

Our finding that perceptions of face ethnicity depended on the raters’ own group membership has both methodological as well as conceptual implications. Methodologically, our data show that what may serve as a typical Indian Asian face in a study with both U.S. and Indian participants is not an equally typical face for both participant groups. Similarly, the manipulation of target group membership or ethnicity through the use of faces (e.g., Krumhuber et al., 2015) may be compromised if the faces end up being misclassified.

Conceptually, our finding that perceptions of face ethnicity depended on the raters’ own group membership is consistent with well-documented effects of familiarity on categorization speed and accuracy (e.g., Smith, 1967; Johnson and Mervis, 1997). However, in the face perception literature, few studies have directly investigated the role of familiarity for the categorization of faces by social group or ethnicity.

Indirect evidence comes from work on the “other-race-effect,” whereby own-race faces are more readily and accurately identified than other-race faces (ORE, see Meissner and Brigham, 2001). One explanation for the ORE holds that face processing occurs along face dimensions that effectively differentiate among the types of faces frequently encountered (Goldstein and Chance, 1980; Valentine, 1991). As a result, more familiar own-race faces function as a perceptual default facilitating their processing and identification, while impeding the processing and identification of other-race faces.

The ORE, thus, is consistent with our finding that ethnicity can be inferred more accurately for ingroup than outgroup faces. However, ORE studies do not directly assess categorization accuracy of the stimulus faces. In fact, studies that do ask participants to classify faces by race, have found the opposite effect, showing that classification is *faster* and *more* accurate for other-race than own-race faces, an effect labeled other-classification race advantage (ORCA; see Levin, 1996; Zhao and Bentin, 2011). Yet, a notable difference between these demonstrations and our current study is that our participants chose from a list of eight ethnic categories, whereas ORCA studies use a category-verification task with a binary choice option (e.g., Asian, Caucasian). In category verification, participants see an array of faces and have to decide whether the face is either Asian or Caucasian. Such a binary choice task is likely to increase the salience of features that differentiate between the two groups used in the task (see Wang et al., 2016).

At times, social interactions may require such a binary differentiation. But often interactions lack explicit group identifiers. With considerable frequency, we encounter people not knowing their ethnic origin, whether they are from the U.S., Europe, India, the Middle East, or any other part of the world. Our data capture the kinds of face impressions people form under these circumstances. We believe research on face impression and stereotyping will benefit from considering a cross-cultural and international context in which the origin of a face is not immediately determined by a small set size of stimulus attributes. We hope the India Face set helps facilitate such research.

The data and materials for this research are available at www.chicagofaces.org.

## Data Availability Statement

The raw data supporting the conclusions of this article will be made available by authors, without undue reservation, to any qualified researchers with verifiable credentials, who do not pose a risk to confidentiality and safety of our participants.

## Ethics Statement

The studies involving human participants were reviewed and approved by the Institutional Review Board, University of Chicago. The patients/participants provided their written/online informed consent to participate in this study. Written/online informed consent was obtained from the individual(s) for the publication of any potentially identifiable images or data included in this article.

## Author Contributions

AL and BW designed and supervised image collection and standardization, designed and conducted the norming study, analyzed data, and co-wrote the manuscript. JC analyzed the data and co-wrote the manuscript. DM co-wrote the manuscript. All authors contributed to the article and approved the submitted version.

## Conflict of Interest

The authors declare that the research was conducted in the absence of any commercial or financial relationships that could be construed as a potential conflict of interest.

## Footnotes

1 Faces with emotional expressions are not of immediate interest to the current study. Their selection and standardization followed the procedures outlined in Ma et al. (2015).

2 For targets with traditional signifiers, like the aforementioned vermillion head marking (sindoor), or a marriage necklace (mangal sutra), we prepared duplicate image versions where technically feasible. Both versions were processed in identical fashion, except version 1 removed the signifiers whereas version 2 kept them intact. Version 1 was used for the norming data collection. But as researchers may be interested in alternate versions of the same target, both are distributed with the CFD-India image set.

3 Our analyses are based on 172 targets of Indian Asian ethnicity. In addition, the India face set includes 12 models of North East Indian Asian ethnicity that were not considered for the current *analyses.*
