# Supplementary material for: The India Face Set: International and Cultural Boundaries Impact Face Impressions and Perceptions of Category Membership
Source: Front Psychol. 2021 Feb 11;12:627678. doi: 10.3389/fpsyg.2021.627678 (PMC7905305; doi:10.3389/fpsyg.2021.627678)
Supplement: Supplementary file 1 [file Data_Sheet_1.pdf]

Supplementary Materials for Lakshmi, A. J., Wittenbrink, B., Correll, J., & Ma, D. S. (2021). The India Face Set: International and Cultural Boundaries Impact Face Impressions and Perceptions of Category Membership. *Frontiers in Psychology, 12*, 161.

1. Materials

CFD image files used as Caucasian targets:

CFD-WF-001-003-N, CFD-WF-002-004-N, CFD-WF-003-003-N, CFD-WF-005-010-N, CFD-WF-007-001-N, CFD-WF-008-002-N, CFD-WF-010-004-N, CFD-WF-011-002-N, CFD-WF-013-003-N, CFD-WF-015-006-N, CFD-WF-016-015-N, CFD-WF-017-003-N, CFD-WF-018-017-N, CFD-WF-019-005-N, CFD-WF-022-017-N, CFD-WF-023-003-N, CFD-WF-024-003-N, CFD-WF-025-019-N, CFD-WF-028-023-N, CFD-WF-034-006-N, CFD-WF-036-023-N, CFD-WF-038-021-N, CFD-WF-201-156-N, CFD-WF-202-056-N, CFD-WF-203-229-N, CFD-WF-207-014-N, CFD-WF-208-068-N, CFD-WF-211-001-N, CFD-WF-212-050-N, CFD-WF-213-031-N, CFD-WF-218-087-N, CFD-WF-222-092-N, CFD-WF-224-099-N, CFD-WF-226-095-N, CFD-WF-227-002-N, CFD-WF-228-196-N, CFD-WF-230-158-N, CFD-WF-231-099-N, CFD-WF-232-161-N, CFD-WF-234-086-N, CFD-WF-237-067-N, CFD-WF-241-210-N, CFD-WF-242-001-N, CFD-WF-243-148-N, CFD-WF-244-163-N, CFD-WF-245-084-N, CFD-WF-246-087-N, CFD-WF-248-129-N, CFD-WF-249-126-N, CFD-WF-250-167-N, CFD-WF-251-014-N, CFD-WF-252-159-N, CFD-WM-001-014-N, CFD-WM-002-009-N, CFD-WM-003-002-N, CFD-WM-004-010-N, CFD-WM-006-002-N, CFD-WM-009-002-N, CFD-WM-010-001-N, CFD-WM-012-001-N, CFD-WM-013-001-N, CFD-WM-014-002-N, CFD-WM-015-002-N, CFD-WM-016-001-N, CFD-WM-017-002-N, CFD-WM-018-002-N, CFD-WM-019-003-N, CFD-WM-020-001-N, CFD-WM-021-001-N, CFD-WM-022-001-N, CFD-WM-023-001-N, CFD-WM-024-015-N, CFD-WM-025-002-N, CFD-WM-026-001-N, CFD-WM-028-003-N, CFD-WM-029-023-N, CFD-WM-031-003-N, CFD-WM-032-001-N, CFD-WM-033-025-N, CFD-WM-034-030-N, CFD-WM-035-032-N, CFD-WM-036-031-N, CFD-WM-037-025-N, CFD-WM-038-003-N, CFD-WM-039-018-N, CFD-WM-041-021-N, CFD-WM-200-034-N, CFD-WM-201-063-N, CFD-WM-202-107-N, CFD-WM-203-023-N, CFD-WM-204-031-N, CFD-WM-205-007-N, CFD-WM-206-045-N, CFD-WM-207-048-N, CFD-WM-208-068-N, CFD-WM-209-038-N, CFD-WM-210-057-N, CFD-WM-211-054-N, CFD-WM-212-097-N, CFD-WM-213-076-N, CFD-WM-214-026-N, CFD-WM-215-041-N, CFD-WM-216-061-N, CFD-WM-217-070-N, CFD-WM-218-074-N, CFD-WM-219-008-N, CFD-WM-220-068-N, CFD-WM-221-091-N, CFD-WM-222-057-N, CFD-WM-223-056-N, CFD-WM-224-197-N, CFD-WM-225-127-N, CFD-WM-228-065-N, CFD-WM-229-129-N, CFD-WM-230-131-N, CFD-WM-231-112-N, CFD-WM-232-070-N, CFD-WM-233-106-N, CFD-WM-234-118-N, CFD-WM-235-147-N, CFD-WM-236-072-N, CFD-WM-237-052-N, CFD-WM-238-020-N, CFD-WM-239-128-N, CFD-WM-240-125-N, CFD-WM-241-072-N, CFD-WM-242-011-N, CFD-WM-243-107-N, CFD-WM-244-003-N, CFD-WM-245-123-N, CFD-WM-247-084-N, CFD-WM-248-036-N, CFD-WM-249-239-N, CFD-WM-250-157-N, CFD-WM-251-002-N, CFD-WM-252-224-N, CFD-WM-253-119-N, CFD-WM-254-152-N, CFD-WM-255-219-N, CFD-WM-256-138-N, CFD-WM-257-161-N, CFD-WM-258-125-N

Table 1

Physical Facial Features and Measurements

Facial Feature	Measurement
Median Luminance	Median luminance of the face without neck or hair
Nose Width	Distance between outside edge of the nose at widest point
Nose Length	Distance between nose tip and upper edge of eyes at nose tip center
Lip Thickness	Distance between top and bottom edge of lips at thickest point
Face Length	Distance between bottom of chin to edge of top of forehead/hairline
Eye Height	Distance between upper and lower inner eyelid at pupil center (right, left, averaged)
Eye Width	Distance between inner and outer corner of eye (right, left, averaged)
Face Width at Cheek	Distance between the outer edges of the cheek at most prominent point
Face Width at Mouth	Distance between outer edges of cheeks at mid-mouth
Bizygomatic Face Width	Maximum distance between left and right facial boundary
Forehead Length	Distance from center of top of forehead/hairline to the center between the eyes at pupils
Distance Between Pupils	Distance between the center of each pupil
Distance Between Pupil and Top of Face	Distance between pupil center to top of forehead/hairline (right, left, averaged)
Distance Between Pupil and Upper Lip	Distance between pupil center to top edge of lips (right, left, averaged)
Chin Length	Distance from bottom edge of lips to base of chin
Length of Cheek to Chin	Distance between midcheek to bottom of chin (right, left, averaged)
Midbrow to Hairline	Distance between middle eyebrow to top of forehead/hairline (right, left, averaged)
Facial Width-to-Height Ratio (fWHR1)	(Face Width at Cheek) ÷ (Distance between upperlip and brow)
Bizygomatic Facial Width-to-Height Ratio (fWHR2)	(Bizygomatic Face Width) ÷ (Upper Face Length)
Face shape	(Face Width at Most Prominent Part of the Cheek) ÷ (Face Length)
Heartshapeness	(Face Width at Most Prominent Part of the Cheek) ÷ (Face Width at Mouth)
Nose shape	(Nose width) ÷ (Nose Length)
Lip Fullness	(Lip thickness) ÷ (Face length)
Eye Shape	(Eye height) ÷ (Eye width)
Eye Size	(Eye height) ÷ (Face length)
Upper Head Length	(Forehead length) ÷ (Face length)
Midface Length	(Distance between pupil and upper lip averaged for right and left side) ÷ (Face length)
Upper Face Length	Distance between the upper lip and highest point of the eyelids
Chin Size	(Chin length) ÷ (Face length)
Forehead Height	(Midbrow to Hairline averaged for right and left side) ÷ (Face length)
Cheekbone Height	(Length of cheek to chin averaged for right and left side) ÷ (Face length)
Cheekbone Prominence	(Face Width at Cheek – Face width at mouth) ÷ (Face Length)
Face Roundness	(Face width at mouth) ÷ (Face length)
Face Color RGB	Median red, green, and blue color channel values of the face without neck or hair
Hair Color RGB	Median red, green, and blue color channel values of the hair
Eyebrow Thickness	Distance between upper and lower edge of eyebrow at pupil (right, left, averaged)
Eyelid Thickness	Distance between upper and lower edge of eyelid at pupil (right, left, averaged)

Note. Measurements were taken using Adobe Photoshop. Distance measures were assessed in pixels.

Table 2

Physical Facial Feature Measurement Reliability with Cronbach's Alpha

Attribute	Alpha	Attribute	Alpha
Luminance_median	1.00	BottomLip_Chin	0.95
Nose_Width	1.00	Midcheek_Chin_Right	0.90
Nose_Length	0.89	Midcheek_Chin_Left	0.90
Lip_Thickness	0.99	Midbrow_Hairline_Right	0.96
Face_Length	0.96	Midbrow_Hairline_Left	0.96
Right_Eye_Height	0.99	FaceColor_Red	1.00
Left_Eye_Height	0.99	FaceColor_Green	1.00
Right_Eye_Width	0.96	FaceColor_Blue	1.00
Left_Eye_Width	0.97	EyeBrow_Thickness_Right	0.94
Face_Width_Cheeks	0.83	EyeBrow_Thickness_Left	0.95
Face_Width_Mouth	0.75	EyeLid_Thickness_Right	1.00
Face_Width_Ears	0.97	EyeLid_Thickness_Left	1.00
Forehead	0.95	Avg_Eye_Height	0.99
Pupil_Top_Right	0.95	Avg_Eye_Width	0.97
Pupil_Top_Left	0.96	Asymmetry_pupil_top	0.57
Pupil_Lip_Right	0.97	Asymmetry_pupil_lip	0.50
Pupil_Lip_Left	0.96	Cheeks_avg	0.91
EyeDistance	0.98	EyeLid_Thickness_avg	1.00
		EyeBrow_Thickness_avg	0.96

2. Results

Table 3

Main Effect of Participant Race: Regression Test Statistics

Predictors	*Estimates*	*CI*	*t*	*df*	*p*	η^2_p
(Intercept)	-0.08	-0.43 – 0.26	-0.49	1961.35	0.625	
pRace_Code	-0.11	-0.75 – 0.52	-0.36	1774.69	0.722	0.00007 | [0.00, 0.00]
tRace_Code	-0.96	-1.23 – -0.69	-7.04	277.32	<0.001	0.15 | [0.09, 0.22]
pRace_Code * tRace_Code	-0.79	-0.99 – -0.59	-7.71	12188.38	<0.001	0.005 | [0.00, 0.01]
N_{Face}	284					
$N_{Subject}$	1778					
Observations	14224					
Marginal R^2 / Conditional R^2	0.005 / 0.835					

Table 4

Individual Impression Attributes: Regression Test Statistics

Attribute	Test Statistics						
Attractive	*Predictors*	*Estimates*	*CI*	*t*	*df*	*p*	η^2_p
	(Intercept)	4.03	3.95 – 4.11	99.51	524.5	<0.001	
	pRace_Code	-0.04	-0.13 – 0.06	-0.76	1765.99	0.449	0.0003 | [0.00, 0.00]
	tRace_Code	-0.36	-0.50 – -0.23	-5.29	280.88	<0.001	0.09 | [0.04, 0.15]
	pRace_Code * tRace_Code	-0.27	-0.35 – -0.19	-6.56	12179.08	<0.001	0.004 | [0.00, 0.01]
	N_{Face}	284					
	$N_{Subject}$	1778					
	Observations	14224					
	Marginal R^2 / Conditional R^2	0.014 / 0.444					
Warm	*Predictors*	*Estimates*	*CI*	*t*	*df*	*p*	η^2_p
	(Intercept)	3.85	3.79 – 3.92	119.72	973.7	<0.001	
	pRace_Code	-0.01	-0.11 – 0.09	-0.19	1770.49	0.846	0.00002 | [0.00, 0.00]
	tRace_Code	-0.05	-0.13 – 0.04	-1.1	269.59	0.27	0.005 | [0.00, 0.03]
	pRace_Code * tRace_Code	-0.06	-0.14 – 0.02	-1.47	12195.76	0.141	0.0002 | [0.00, 0.00]
	N_{Face}	284					
	$N_{Subject}$	1778					
	Observations	14224					
	Marginal R^2 / Conditional R^2	0.000 / 0.439					

Competent	*Predictors*	*Estimates*	*CI*	*t*	*df*	*p*	η^2_p
	(Intercept)	4.36	4.30 – 4.41	153.71	1199.22	<0.001	
	pRace_Code	-0.42	-0.52 – -0.33	-8.83	1770.23	<0.001	0.04 | [0.03, 0.06]
	tRace_Code	-0.01	-0.08 – 0.06	-0.25	265.51	0.8	0.0002 | [0.00, 0.01]
	pRace_Code * tRace_Code	-0.15	-0.22 – -0.09	-4.45	12199.44	<0.001	0.002 | [0.00, 0.00]
	N_{Face}	284					
	$N_{Subject}$	1778					
	Observations	14224					
	Marginal R^2 / Conditional R^2	0.022 / 0.483					
Trustworthy	*Predictors*	*Estimates*	*CI*	*t*	*df*	*p*	η^2_p
	(Intercept)	4.3	4.24 – 4.36	145.86		<0.001	
	pRace_Code	-0.03	-0.12 – 0.06	-0.61		0.544	0.0003 | [0.00, 0.00]
	tRace_Code	0.01	-0.07 – 0.09	0.25		0.804	0.0003 | [0.00, 0.00]
	pRace_Code * tRace_Code	-0.08	-0.15 – -0.01	-2.2		0.028	0.0004 | [0.00, 0.00]
	N_{Face}	284					
	$N_{Subject}$	1778					
	Observations	14224					
	Marginal R^2 / Conditional R^2	0.000 / 0.431					
Happy	*Predictors*	*Estimates*	*CI*	*t*	*df*	*p*	η^2_p
	(Intercept)	3.36	3.27 – 3.45	74.54	651.13	<0.001	
	pRace_Code	0.19	0.07 – 0.30	3.24	1769.91	0.001	0.006 | [0.00, 0.01]
	tRace_Code	-0.22	-0.36 – -0.08	-3.06	277.42	0.002	0.03 | [0.01, 0.07]
	pRace_Code * tRace_Code	-0.04	-0.12 – 0.03	-1.13	12173.58	0.258	0.0001 | [0.00, 0.00]
	N_{Face}	284					
	$N_{Subject}$	1778					
	Observations	14224					
	Marginal R^2 / Conditional R^2	0.007 / 0.549					
Angry	*Predictors*	*Estimates*	*CI*	*t*	*df*	*p*	η^2_p
	(Intercept)	2.87	2.79 – 2.95	69.24	874.12	<0.001	
	pRace_Code	-0.18	-0.30 – -0.06	-2.89	1770.17	0.004	0.005 | [0.00, 0.01]
	tRace_Code	0.07	-0.05 – 0.19	1.16	270.99	0.246	0.005 | [0.00, 0.03]
	pRace_Code * tRace_Code	-0.08	-0.16 – -0.00	-2.03	12178.39	0.043	0.0003 | [0.00, 0.00]
	N_{Face}	284					
	$N_{Subject}$	1778					
	Observations	14224					
	Marginal R^2 / Conditional R^2	0.003 / 0.540					

	Predictors	Estimates	CI	t	df	p	η^2_p
Sad	*Predictors*	*Estimates*	*CI*	*t*	*df*	*p*	η^2_p
	(Intercept)	3.18	3.10 – 3.27	73.38	697.58	<0.001	
	pRace_Code	0.06	-0.06 – 0.17	0.96	1767.49	0.336	0.0005 | [0.00, 0.00]
	tRace_Code	0.15	0.01 – 0.28	2.17	279.11	0.03	0.02 | [0.00, 0.05]
	pRace_Code * tRace_Code	0.06	-0.03 – 0.14	1.29	12177.72	0.197	0.001
	N_{Face}	284					
	$N_{Subject}$	1778					
	Observations	14224					
	Marginal R^2 / Conditional R^2	0.002 / 0.490					
Disgusted	*Predictors*	*Estimates*	*CI*	*t*	*df*	*p*	η^2_p
	(Intercept)	2.77	2.69 – 2.84	74.41	1533.71	<0.001	
	pRace_Code	0.02	-0.11 – 0.15	0.28	1771.83	0.782	0.00004 | [0.00, 0.00]
	tRace_Code	0.06	-0.02 – 0.14	1.43	267.22	0.153	0.008 | [0.00, 0.03]
	pRace_Code * tRace_Code	-0.06	-0.13 – 0.02	-1.53	12197.78	0.126	0.0002 | [0.00, 0.00]
	N_{Face}	284					
	$N_{Subject}$	1778					
	Observations	14224					
	Marginal R^2 / Conditional R^2	0.000 / 0.595					
Surprised	*Predictors*	*Estimates*	*CI*	*t*	*df*	*p*	η^2_p
	(Intercept)	2.71	2.64 – 2.78	73.61	1855.89	<0.001	
	pRace_Code	0.39	0.25 – 0.52	5.51	1773.89	<0.001	0.02 | [0.01, 0.03]
	tRace_Code	-0.12	-0.18 – -0.07	-4.31	283.29	<0.001	0.06 | [0.02, 0.11]
	pRace_Code * tRace_Code	-0.04	-0.11 – 0.02	-1.23	12234.27	0.221	0.0001 | [0.00, 0.00]
	N_{Face}	284					
	$N_{Subject}$	1778					
	Observations	14224					
	Marginal R^2 / Conditional R^2	0.013 / 0.678					
Fearful	*Predictors*	*Estimates*	*CI*	*t*	*df*	*p*	η^2_p
	(Intercept)	2.81	2.74 – 2.88	74.67	1405.73	<0.001	
	pRace_Code	0.26	0.14 – 0.39	4.11	1771.83	<0.001	0.010 | [0.00, 0.02]
	tRace_Code	0.07	-0.02 – 0.15	1.53	270.67	0.126	0.009 | [0.00, 0.04]
	pRace_Code * tRace_Code	-0.05	-0.13 – 0.02	-1.42	12194.46	0.156	0.0002| [0.00, 0.00]
	N_{Face}	284					
	$N_{Subject}$	1778					
	Observations	14224					
	Marginal R^2 / Conditional R^2	0.006 / 0.586					

	Predictors	Estimates	CI	t	df	p	η^2_p
Threatening	*Predictors*	*Estimates*	*CI*	*t*	*df*	*p*	η^2_p
	(Intercept)	2.99	2.92 – 3.07	76.71	1066.33	<0.001	
	pRace_Code	0.04	-0.08 – 0.17	0.72	1770.54	0.474	0.0003 | [0.00, 0.00]
	tRace_Code	0.04	-0.06 – 0.14	0.78	268.31	0.437	0.003 | [0.00, 0.02]
	pRace_Code * tRace_Code	-0.01	-0.09 – 0.07	-0.29	12185.64	0.775	0.000007 | [0.00, 0.00]
	N_{Face}	284					
	$N_{Subject}$	1778					
	Observations	14224					
	Marginal R^2 / Conditional R^2	0.000 / 0.523					
Masculine	*Predictors*	*Estimates*	*CI*	*t*	*df*	*p*	η^2_p
	(Intercept)	4.18	4.03 – 4.33	53.52	308.37	<0.001	
	pRace_Code	-0.18	-0.26 – -0.10	-4.41	1757.41	<0.001	0.01 | [0.00, 0.02]
	tRace_Code	0.13	-0.17 – 0.43	0.86	283.77	0.389	0.003 | [0.00, 0.02]
	pRace_Code * tRace_Code	-0.1	-0.20 – -0.01	-2.13	12175.81	0.034	0.0004 | [0.00, 0.00]
	N_{Face}	284					
	$N_{Subject}$	1778					
	Observations	14224					
	Marginal R^2 / Conditional R^2	0.003 / 0.493					
Feminine	*Predictors*	*Estimates*	*CI*	*t*	*df*	*p*	η^2_p
	(Intercept)	3.41	3.23 – 3.59	37.89	300.99	<0.001	
	pRace_Code	0.01	-0.07 – 0.08	0.16	1757.34	0.877	0.00001 | [0.00, 0.00]
	tRace_Code	-0.23	-0.58 – 0.12	-1.29	283.28	0.198	0.006 | [0.00, 0.03]
	pRace_Code * tRace_Code	0.02	-0.07 – 0.11	0.39	12174.11	0.699	0.00001 | [0.00, 0.00]
	N_{Face}	284					
	$N_{Subject}$	1778					
	Observations	14224					
	Marginal R^2 / Conditional R^2	0.003 / 0.576					
Babyfaced	*Predictors*	*Estimates*	*CI*	*t*	*df*	*p*	η^2_p
	(Intercept)	3.12	3.04 – 3.21	71.38	695.64	<0.001	
	pRace_Code	-0.4	-0.52 – -0.29	-6.84	1768.46	<0.001	0.03 | [0.01, 0.04]
	tRace_Code	-0.52	-0.65 – -0.38	-7.58	278.78	<0.001	0.17 | [0.11, 0.24]
	pRace_Code * tRace_Code	-0.13	-0.21 – -0.04	-2.91	12177.93	0.004	0.0007 | [0.00, 0.00]
	N_{Face}	284					
	$N_{Subject}$	1778					
	Observations	14224					
	Marginal R^2 / Conditional R^2	0.032 / 0.510					

Unusual	*Predictors*	*Estimates*	*CI*	*t*	*df*	*p*	η^2_p
	(Intercept)	3.09	3.03 – 3.16	88.33	1591.09	<0.001	
	pRace_Code	0.09	-0.04 – 0.21	1.36	1771.9	0.173	0.001 | [0.00, 0.01]
	tRace_Code	0.11	0.04 – 0.17	3.1	251.18	0.002	0.04 | [0.01, 0.08]
	pRace_Code * tRace_Code	-0.38	-0.46 – -0.30	-9.2	12203.21	<0.001	0.007 | [0.00, 0.01]
	N_{Face}	284					
	$N_{Subject}$	1778					
	Observations	14224					
	Marginal R^2 / Conditional R^2	0.004 / 0.541					
Status	*Predictors*	*Estimates*	*CI*	*t*	*df*	*p*	η^2_p
	(Intercept)	4.42	4.36 – 4.48	144.86	885.59	<0.001	
	pRace_Code	0.31	0.24 – 0.38	8.52	1764.27	<0.001	0.04 | [0.03, 0.06]
	tRace_Code	-0.48	-0.58 – -0.38	-9.33	276.33	<0.001	0.24 | [0.17, 0.31]
	pRace_Code * tRace_Code	-0.28	-0.34 – -0.21	-8.28	12170.35	<0.001	0.006 | [0.00, 0.01]
	N_{Face}	284					
	$N_{Subject}$	1778					
	Observations	14224					
	Marginal R^2 / Conditional R^2	0.051 / 0.421					

Table 5

Stereotype Content: Regression Test Statistics

Attribute	Test Statistics						
Warm	*Predictors*	*Estimates*	*CI*	*t*	*df*	*p*	η^2_p
	(Intercept)	3.85	3.79 – 3.92	119.72	973.75	<0.001	
	tRace_Code	-0.05	-0.13 – 0.04	-1.1	269.59	0.27	0.005 | [0.00, 0.03]
	pRace_Code	-0.01	-0.11 – 0.09	-0.19	1770.49	0.846	0.00002 | [0.00, 0.00]
	tRace_Code * pRace_Code	-0.06	-0.14 – 0.02	-1.47	12195.76	0.141	0.0002 | [0.00, 0.00]
	N_{Face}	284					
	$N_{Subject}$	1778					
	Observations	14224					
	Marginal R^2 / Conditional R^2	0.000 / 0.439					
Competent	*Predictors*	*Estimates*	*CI*	*t*	*df*	*p*	η^2_p
	(Intercept)	4.36	4.30 – 4.41	153.71	1199.22	<0.001	
	tRace_Code	-0.01	-0.08 – 0.06	-0.25	265.51	0.8	0.0002 | [0.00, 0.01]
	pRace_Code	-0.42	-0.52 – -0.33	-8.83	1770.23	<0.001	0.04 | [0.03, 0.06]
	tRace_Code * pRace_Code	-0.15	-0.22 – -0.09	-4.45	12199.44	<0.001	0.002 | [0.00, 0.00]
	N_{Face}	284					
	$N_{Subject}$	1778					
	Observations	14224					
	Marginal R^2 / Conditional R^2	0.022 / 0.483					
Trustworthy	*Predictors*	*Estimates*	*CI*	*t*	*df*	*p*	η^2_p
	(Intercept)	4.3	4.24 – 4.36	145.86	885.59	<0.001	
	tRace_Code	0.01	-0.07 – 0.09	0.25	266.83	0.804	0.0002 | [0.00, 0.01]
	pRace_Code	-0.03	-0.12 – 0.06	-0.61	1768.2	0.544	0.0002| [0.00, 0.00]
	tRace_Code * pRace_Code	-0.08	-0.15 – -0.01	-2.2	12185.92	0.028	0.0004 | [0.00, 0.00]
	N_{Face}	284					
	$N_{Subject}$	1778					
	Observations	14224					
	Marginal R^2 / Conditional R^2	0.000 / 0.431					

Table 6

Ingroup Favoritsm: Regression Test Statistics

Predictors	*Estimates*	*CI*	*t*	*df*	*p*	*peta*
(Intercept)	1.05	0.96 – 1.15	21.86	482.29	**<0.001**	
tRace_Code	-0.2	-0.36 – -0.04	-2.41	278.6	**0.016**	0.02 | [0.00, 0.06]
pRace_Code	-0.1	-0.20 – -0.00	-1.96	1766.77	**0.05**	2.18e-03 | [0.00, 0.01]
tRace_Code * pRace_Code	-0.09	-0.17 – -0.01	-2.24	12171.45	**0.025**	4.13e-04 | [0.00, 0.00]
N_{Face}	284					
$N_{Subject}$	1778					
Observations	14224					
Marginal R^2 / Conditional R^2	0.004 / 0.506					

3. Data Counts and Power Analyses

For studies in which a sample of participants rates a sample of targets, power is a function of both the number of participants and the number of targets. We did not conduct an a priori power analysis for the current study, but we collected data from both a large number of participants (n= 850+ per participant group for trait ratings and categorization decisions, n = 200+ per participant group for typicality ratings) and a large number of targets (m = 142 per target group), which should yield relatively high power (Westfall, Kenny, & Judd, 2014). In some ways, it would be advantageous to have each participant rate each target, but given the large number of targets, that approach was impractical. Each participant therefore rated a small proportion of the full set of targets, which yields a fairly sparse array with data missing at random. This approach actually preserves power fairly well because it retains a large number of participants and a large number of targets (Judd, Westfall, & Kenny, 2017).

Because data were missing at random, we have some targets that were rated more often than others. Individual Indian face stimuli were rated by 13- 40 same group participants and 15-39 other group participants. Individual U.S. face stimuli were rated by 14- 39 same group participants and 15-40 other group participants. To address a reviewer's concern about the impact of this on power, we conducted a post-hoc sensitivity analysis using the R package, simr (Baayen, Davidson, & Bates, 2008) on our mixed effects model analyses. This package requires an existing multilevel model. The estimates of the fixed and random effects of that model can be used to perform Monte Carlo simulations (simulating a new dataset based on the model estimates) and compute power for a range of unstandardized effect sizes. The advantage of this approach is that it uses the actual structure of these data (including the unequal presentation of targets) when estimating power.

Our sensitivity analysis for trait ratings was based on the results of 5 different randomly selected mixed-effects models, a subset of the analyses we have reported. Each model was used to perform the simulations, and we computed the power to detect interactions that covered the full range of the effects that we observed in the analyses (ranging from zero to the largest effect,

b=0.37). On average, this analysis had adequate power (.80) to detect interactions as small as b=0.15 (see Figure 1). Since this approach depends on the manner in which variables are measured, we conducted separate sensitivity analysis for typicality ratings – the study had adequate power (.80) to detect interactions as small as b=0.12).

Figure 1

Sensitivity Analysis for Trait Rating Models

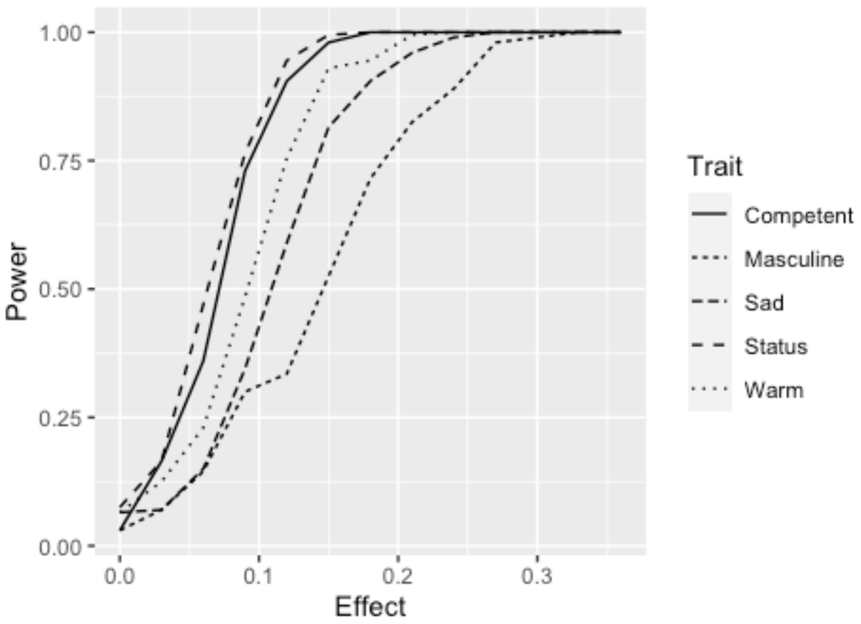

References

Judd, C. M., Westfall, J., & Kenny, D. A. (2017). Experiments with more than one random factor: Designs, analytic models, and statistical power. *Annual Review of Psychology, 68*, 601-625.

Baayen, R. H., Davidson, D. J., & Bates, D. M. (2008). Mixed-effects modeling with crossed random effects for subjects and items. *Journal of memory and language, 59(4)*, 390-412.
